# A computationally efficient algorithm to leverage average information REML for (co)variance component estimation in the genomic era

**DOI:** 10.1186/s12711-024-00939-x

**Published:** 2024-11-21

**Authors:** Ismo Strandén, Esa A. Mäntysaari, Martin H. Lidauer, Robin Thompson, Hongding Gao

**Affiliations:** 1 Natural Resources Institute Finland (Luke), 31600 Jokioinen, Finland; 2 Rothamsted Research, Harpenden, Herts AL5 2JQ UK

## Abstract

**Background:**

Methods for estimating variance components (VC) using restricted maximum likelihood (REML) typically require elements from the inverse of the coefficient matrix of the mixed model equations (MME). As genomic information becomes more prevalent, the coefficient matrix of the MME becomes denser, presenting a challenge for analyzing large datasets. Thus, computational algorithms based on iterative solving and Monte Carlo approximation of the inverse of the coefficient matrix become appealing. While the standard average information REML (AI-REML) is known for its rapid convergence, its computational intensity imposes limitations. In particular, the standard AI-REML requires solving the MME for each VC, which can be computationally demanding, especially when dealing with complex models with many VC. To bridge this gap, here we (1) present a computationally efficient and tractable algorithm, named the augmented AI-REML, which facilitates the AI-REML by solving an augmented MME only once within each REML iteration; and (2) implement this approach for VC estimation in a general framework of a multi-trait GBLUP model. VC estimation was investigated based on the number of VC in the model, including a two-trait, three-trait, four-trait, and five-trait GBLUP model. We compared the augmented AI-REML with the standard AI-REML in terms of computing time per REML iteration. Direct and iterative solving methods were used to assess the advances of the augmented AI-REML.

**Results:**

When using the direct solving method, the augmented AI-REML and the standard AI-REML required similar computing times for models with a small number of VC (the two- and three-trait GBLUP model), while the augmented AI-REML demonstrated more notable reductions in computing time as the number of VC in the model increased. When using the iterative solving method, the augmented AI-REML demonstrated substantial improvements in computational efficiency compared to the standard AI-REML. The elapsed time of each REML iteration was reduced by 75%, 84%, and 86% for the two-, three-, and four-trait GBLUP models, respectively.

**Conclusions:**

The augmented AI-REML can considerably reduce the computing time within each REML iteration, particularly when using an iterative solver. Our results demonstrate the potential of the augmented AI-REML as an appealing approach for large-scale VC estimation in the genomic era.

## Background

Accurate estimates of (co)variance components (VC) are crucial in computing precise genetic and genomic predictions. In the context of plant breeding, VC are typically estimated within each cycle of genomic prediction. Conversely, in animal breeding, VC used for genomic prediction are often initially estimated using an animal model with pedigree information and subsequently updated at regular intervals. Although unbiased estimates of VC can be obtained using complete data and a pedigree-based model, results from earlier studies have shown that ignoring the genomic information for populations undergoing intense genomic selection yielded biased estimates of VC [1, 2]. Notably, results from the U.S. dairy cattle genomic evaluation showed that prediction bias decreased when heritability was reduced by about 50% to 70%, indicating an overestimated heritability [3, 4]. Given these findings, there is a growing consensus that the VC estimation needs to be done properly in the genomic era [5, 6]. Consequently, continued attention and refinement are essential for accurate VC estimation.

Restricted maximum likelihood (REML) serves as an important method in genetic analysis. It facilitates VC estimation within multivariate linear mixed models, which are commonly employed in the fields of animal and plant breeding to account for genetic correlations between traits and to make better use of available information across traits. Unlike maximum likelihood estimation, which estimates parameters using the likelihood function, REML adjusts the likelihood function using error contrasts. This adjustment accounts for the loss of degrees of freedom associated with estimating the fixed effects [7]. By using error contrasts, REML produces less biased estimates of VC, making it a widely applied approach in animal and plant breeding [5, 8–10].

Various approaches are available for maximizing the REML likelihood. The two most widely used methods are the expectation–maximization REML (EM-REML) and the average information REML (AI-REML) [11, 12]. EM-REML relies on the first derivatives of the REML log-likelihood, which make it straightforward to implement but suffers from slow convergence. In contrast, AI-REML uses both the first and second derivatives of the REML log-likelihood, resulting in significantly quicker convergence rates [13]. All Newton-type methods such as AI-REML use the vector of first derivatives at the current estimates, along with a matrix that characterizes the information content of the unknown VC in the analysis. The popularity of AI-REML is attributed to its quick convergence compared to the EM-REML; however, AI-REML is computationally more intensive [12, 13].

The analytical REML-based methods, typically used for VC estimation, require elements from the inverse coefficient matrix of the mixed model equations (MME), i.e., the prediction error (co)variances (PEV/PEC). In analyses using pedigree-based models, computations can be efficiently conducted using sparse matrix techniques due to the sparse nature of the coefficient matrix of the MME [14]. However, with the increase of genomic information and the emergence of high-dimensional datasets, the coefficient matrix of the MME tends to become denser. Consequently, animal and plant breeders working with large genomic data need to address this challenge of increased computational complexity.

To improve the computational efficiency and capability of REML analyses, various methods have been introduced. Masuda et al. [15] employed the supernodal methods to optimize the MME setting-up and trace computation in the AI-REML for genomic models and demonstrated that significant performance improvement was achieved compared to the original AI-REML. Recently, Meyer [16] found that, with principal components parameterization of the MME, the computing time of (co)variance components estimation for single-step genomic best linear unbiased prediction (ssGBLUP) using AI-REML can be substantially reduced. Matilainen et al. [17, 18] presented and implemented Monte Carlo (MC) approaches within the EM-REML and Newton-type methods. These approaches allowed the approximation of PEV/PEC without explicitly making or inverting the MME coefficient matrix. Instead, the approximated PEV/PEC were computed by generating MC samples from distributions identical to those of the original data and the current VC estimates. They showed that the MC-based REML methods using the preconditioned conjugate gradient (PCG) solver and the iteration on data method, is an efficient approach for VC estimation in large and complex models.

In the standard AI-REML framework, the Hessian matrix is replaced by an average information (AI) matrix, which is computed as the mean of both the observed and expected information matrices. This approach is widely adopted due to its simplicity, as it eliminates the need for intricate trace calculations required in both the observed and the expected information matrices. Instead, the AI matrix can be computed by solving the MME for each VC, with the data replaced by a working vector derived from the current random effect solutions [13]. However, this step can be computationally intensive for large systems (e.g. multi-trait random regression models), as it requires the construction of a work matrix obtained by repeatedly solving the MME with different right-hand sides (RHS). Thompson [19] identified this issue and proposed an alternative approach, which only requires solving an augmented MME to obtain essential values for AI-REML.

In this study, we highlight the importance of the augmented AI-REML method, as previously proposed by Thompson [19]. In particular, we demonstrate the advantages of the augmented AI-REML over the standard AI-REML. Unlike the standard AI-REML, the augmented AI-REML can bypass the need to construct the working matrix (hereinafter referred to as the **T** matrix) and provide a more flexible framework to achieve the same information matrix with reduced computational cost. Furthermore, we illustrate how the augmented AI-REML differs from the standard AI-REML in the way it updates the VC estimates during each iteration (prototype code provided). We investigate the conditions under which the augmented AI-REML can significantly reduce computational requirements compared to the standard AI-REML. In addition, we explore different solving strategies (both direct and iterative) within the AI-REML framework to enhance computational efficiency. Therefore, the aims of this study were: (1) to present a computationally efficient algorithm of augmented AI-REML, which streamlines the VC estimation by solving an augmented MME only once per AI-REML iteration; and (2) to apply this approach in VC estimation in a general framework for a multi-trait GBLUP model.

## Methods

### Statistical model

Consider a multi-trait GBLUP model [20] for *l* traits:

1 $$\mathbf{y}=\mathbf{X}\mathbf{b}+\mathbf{Z}\mathbf{u}+\mathbf{e}$$

where **y** is the vector of observations for the *l* traits, with *n* records for each trait, **b** is the vector of fixed effects, **u** is the vector of random genomic breeding values, and **e** is the vector of random residuals. The design matrices **X** and **Z** relate the observations to the fixed and random effects, respectively. The random effects **u** and **e** are assumed to be independent of each other: $\mathbf{u} \sim N(\boldsymbol{0}, \mathbf{G})$ and $\mathbf{e} \sim N(\boldsymbol{0}, \mathbf{R})$, where $\mathbf{G}={\mathbf{G}}_{0}\otimes {\mathbf{G}}_{\text{r}m}$, ${\mathbf{G}}_{0}$ is an *l* × *l* genetic variance covariance matrix, **G**_rm_ is a *q* × *q* marker-based genomic relationship matrix [20] with *q* equal to the number of genotyped individuals, $\mathbf{R}={\mathbf{R}}_{0}\otimes \mathbf{I}$, ${\mathbf{R}}_{0}$ is a *l* × *l* residual variance covariance matrix, assuming all traits are recorded for each individual, **I** is an identity matrix size of *n*, and $\otimes$ denotes the Kronecker product. We have assumed that all traits are observed for an individual. Furthermore, we have also assumed that all records to have the same residual covariance structure, i.e., a homogenous variance over individuals. These assumptions can easily be relaxed, but would unnecessarily complicate the following derivations.

When the (co)variance matrices **G**_0_ and **R**_0_ are known, the fixed and random effects can be solved using the MME as follows:

2 $$\left[\begin{array}{cc}\mathbf{X}\mathbf{^{\prime}}{\mathbf{R}}^{-1}\mathbf{X}& \mathbf{X}\mathbf{^{\prime}}{\mathbf{R}}^{-1}\mathbf{Z}\\ {\mathbf{Z}}^{\mathbf{^{\prime}}}{\mathbf{R}}^{-1}\mathbf{X}& {\mathbf{Z}}^{\mathbf{^{\prime}}}{\mathbf{R}}^{-1}\mathbf{Z}+{\mathbf{G}}^{-1}\end{array}\right]\left[\begin{array}{c}\widehat{\mathbf{b}}\\ \widehat{\mathbf{u}}\end{array}\right]=\left[\begin{array}{c}{\mathbf{X}}^{\prime}{\mathbf{R}}^{-1}\mathbf{y}\\ {\mathbf{Z}}^{\mathbf{^{\prime}}}{\mathbf{R}}^{-1}\mathbf{y}\end{array}\right]$$

Let **C** be the coefficient matrix on the left-hand side (LHS) of the MME (2), **s** be the vector of fixed and random effects, i.e., $$\mathbf{s}\mathbf{^{\prime}}=\left[\begin{array}{cc}\mathbf{b}\mathbf{^{\prime}}& \mathbf{u}\mathbf{^{\prime}}\end{array}\right]$$, and $$\mathbf{W}=\left[\begin{array}{cc}\mathbf{X}& \mathbf{Z}\end{array}\right]$$. Let *n*_s_ be the total number of effects in **s**. Then, the MME (2) can be written as $\mathbf{C}\widehat{\mathbf{s}}={\mathbf{W}}^{\mathbf{^{\prime}}}{\mathbf{R}}^{-1}\mathbf{y}$. Denote the vector of unknown VC by the parameter vector $${{\varvec{\uptheta}}}^{\prime} = \left[ {\begin{array}{*{20}c} {{{\varvec{\uptheta}}}_{{\text{G}}}^{\prime} } & {{{\varvec{\uptheta}}}_{{\text{R}}}^{\prime} } \\ \end{array} } \right]$$ where ${{\varvec{\uptheta}}}_{\text{G}}=vech({\mathbf{G}}_{0})$, ${{\varvec{\uptheta}}}_{\text{R}}=vech({\mathbf{R}}_{0})$, and $vech(\times )$ represents the operator extracting the unique elements from a symmetric matrix and reshape them into a vector form. In our case, the **θ** vector of VC has *v* elements, containing *l*(*l* + 1)/2 unique elements from ${\mathbf{G}}_{0}$ and ${\mathbf{R}}_{0}$. Henceforth, we denote the *i*-th genetic (co)variance in **θ**_G_ as ${{\varvec{\uptheta}}}_{{\text{G}}_{i}}$ and the *i*-th residual (co)variance in **θ**_R_ as ${{\varvec{\uptheta}}}_{{\text{R}}_{i}}$, respectively.

### The standard AI REML

The REML log-likelihood function [21] can be written as follows:

3 $$\text{log}\mathcal{L}\left({\varvec{\uptheta}}|\mathbf{y}\right)=const-\frac{1}{2}\text{log}\left|\mathbf{V}\right|-\frac{1}{2}\text{log}\left|{\mathbf{X}}^{\prime}{\mathbf{V}}^{-1}\mathbf{X}\right|-\frac{1}{2}{\mathbf{y}}^{\prime}\mathbf{P}\mathbf{y}$$

where $\mathbf{P}= {\mathbf{R}}^{-1}-{\mathbf{R}}^{-1}\mathbf{W}{\mathbf{C}}^{-1}{\mathbf{W}}^{\prime}{\mathbf{R}}^{-1}$, $\mathbf{V}=\mathbf{Z}\mathbf{G}{\mathbf{Z}}^{\mathbf{^{\prime}}}+\mathbf{R}$, and the constant *const* is independent of VC in** θ**. The REML estimates of $\widehat{{\varvec{\uptheta}}}$ maximize the REML likelihood function $\text{log}\mathcal{L}\left({\varvec{\uptheta}}|\mathbf{y}\right)$ given the observed data.

Because complex models do not allow a closed-form solution of **θ** that maximizes $\text{log}\mathcal{L}\left({\varvec{\uptheta}}|\mathbf{y}\right)$, iterative methods need to be used. The AI-REML [11, 12] updates VC estimates from iteration *k*-1 to iteration *k* using the formula:

4 $$\begin{aligned}{\widehat{{\varvec{\uptheta}}}}^{\left[k\right]}=&{\widehat{{\varvec{\uptheta}}}}^{\left[k-1\right]}+{\varvec{\Delta}}\\=& {\widehat{{\varvec{\uptheta}}}}^{\left[k-1\right]}-{\left[{\mathbf{I}}_{\text{A}}\left({\widehat{{\varvec{\uptheta}}}}^{\left[k-1\right]}\right)\right]}^{-1}\mathbf{J}\left({\widehat{{\varvec{\uptheta}}}}^{\left[k-1\right]}\right)\end{aligned}$$

where ${\widehat{{\varvec{\uptheta}}}}^{[k-1]}$ is the vector of current VC estimates, ${\varvec{\Delta}}$ is the updating vector of VC estimates, ${\mathbf{I}}_{\text{A}}\left({\widehat{{\varvec{\uptheta}}}}^{[k-1]}\right)$ is the AI matrix at ${\widehat{{\varvec{\uptheta}}}}^{[k-1]}$, and $\mathbf{J}\left({\widehat{{\varvec{\uptheta}}}}^{[k-1]}\right)$ is the vector of first derivatives of the REML log-likelihood (aka the gradient vector) with respect to **θ** evaluated at ${\widehat{{\varvec{\uptheta}}}}^{[k-1]}$. The AI matrix is

5 $${\mathbf{I}}_{\text{A}}\left({\varvec{\uptheta}}\right)=\frac{1}{2}\left({\mathbf{I}}_{\text{O}}\left({\varvec{\uptheta}}\right)+{\mathbf{I}}_{\text{E}}\left({\varvec{\uptheta}}\right)\right)$$

where ${\mathbf{I}}_{\text{O}}\left({\varvec{\uptheta}}\right)$ is the observed information matrix and ${\mathbf{I}}_{\text{E}}\left({\varvec{\uptheta}}\right)$ is the expectation of the observed information matrix. Element (*i*,*j*), *i*,*j* = 1,…,*v*, of ${\mathbf{I}}_{\text{O}}\left({\varvec{\uptheta}}\right)$ is

6 $$\frac{{\partial }^{2}\text{log}\mathcal{L}\left({\varvec{\uptheta}}|\mathbf{y}\right)}{\partial {{\varvec{\uptheta}}}_{i}\partial {{\varvec{\uptheta}}}_{j}}={\mathbf{y}}^{\prime}\mathbf{P}\frac{\partial \mathbf{V}}{\partial {{\varvec{\uptheta}}}_{i}}\mathbf{P}\frac{\partial \mathbf{V}}{\partial {{\varvec{\uptheta}}}_{j}}\mathbf{P}\mathbf{y}-\frac{1}{2}tr(\mathbf{P}\frac{\partial \mathbf{V}}{\partial {{\varvec{\uptheta}}}_{i}}\mathbf{P}\frac{\partial \mathbf{V}}{\partial {{\varvec{\uptheta}}}_{j}})$$

where *tr*( ×) represents the matrix trace operator. Element (*i*,*j*), *i*,*j* = 1,…,*v*, of ${\mathbf{I}}_{\text{E}}\left({\varvec{\uptheta}}\right)$ is

7 $$E\left[\frac{{\partial }^{2}\text{log}\mathcal{L}\left({\varvec{\uptheta}}|\mathbf{y}\right)}{\partial {{\varvec{\uptheta}}}_{i}\partial {{\varvec{\uptheta}}}_{j}}\right]=\frac{1}{2}tr\left(\mathbf{P}\frac{\partial \mathbf{V}}{\partial {{\varvec{\uptheta}}}_{i}}\mathbf{P}\frac{\partial \mathbf{V}}{\partial {{\varvec{\uptheta}}}_{j}}\right)$$

**Fig. 1:**
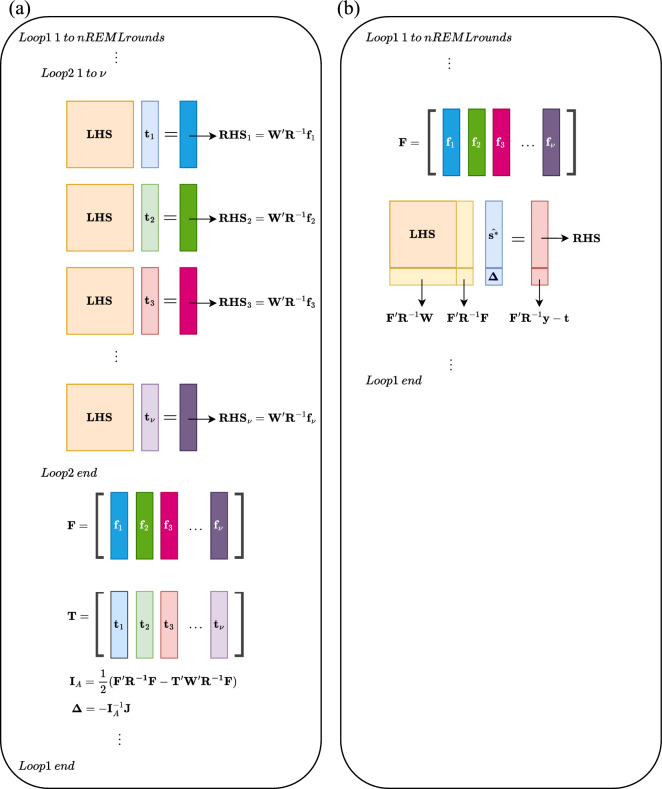
Illustration of the standard AI-REML. **a** vs. the augmented AI-REML **b** for (co)variance component (VC) estimation. The standard AI-REML requires solving the mixed model equations (MME) for each VC to obtain the working vector (${\mathbf{t}}_{i=1,\cdots ,\nu }$), with the right-hand side (RHS) replaced by a suitable working vector (${\mathbf{f}}_{i=1,\cdots ,\nu }$), where n is the total number of VC in the model, $$\mathbf{W}=\left[\begin{array}{cc}\mathbf{X}& \mathbf{Z}\end{array}\right]$$, $\mathbf{R}={\mathbf{R}}_{0}\otimes \mathbf{I}$, ${\mathbf{R}}_{0}$ is a *l* × *l* residual variance covariance matrix, assuming all traits (*l*) are recorded for each individual, **I** is an identity matrix size of *n*/*l*, with *n* equal to the number of observations, and $\otimes$ denotes the Kronecker product. Note that the left-hand side (LHS) of the MME is unchanged for each solving process. **F** and **T** are working matrices containing column vectors of ${\mathbf{f}}_{i=1,\cdots ,\nu }$ and ${\mathbf{t}}_{i=1,\cdots ,\nu }$, respectively. The average information matrix ${\mathbf{I}}_{\text{A}}$ is computed using **F** and **T**, then the updating vector based on the current VC estimates (${\varvec{\Delta}}$) is computed using the inverse of ${\mathbf{I}}_{\text{A}}$ and gradient vector (**J**). For the augmented AI-REML, the updating vector **Δ** can be solved using an MME where the original MME and model has been augmented by an effect **Δ** with the working matrix **F** and RHS for this effect has been corrected by the trace terms (**t**) in the first derivatives of the REML log-likelihood

Hence, element (*i*,*j*) of the AI matrix is $\frac{1}{2}{\mathbf{y}}^{\prime}\mathbf{P}\frac{\partial \mathbf{V}}{\partial {{\varvec{\uptheta}}}_{i}}\mathbf{P}\frac{\partial \mathbf{V}}{\partial {{\varvec{\uptheta}}}_{j}}\mathbf{P}\mathbf{y}$ [11, 13] and the AI matrix is

8 $$\begin{aligned}{\mathbf{I}}_{\text{A}}\left({\varvec{\uptheta}}\right)=&\frac{1}{2}{\mathbf{F}}^{{^{\prime}}}\mathbf{P}\mathbf{F}\\=&\frac{1}{2}\left({\mathbf{F}}^{{\prime}}{\mathbf{R}}^{-1}\mathbf{F}-{{\mathbf{F}}^{{\prime}}{\mathbf{R}}^{-1}\mathbf{W} \mathbf{C}}^{-1}{\mathbf{W}}^{{\prime}}{\mathbf{R}}^{-1}\mathbf{F}\right)\\=&\frac{1}{2}(\mathbf{F}^{\prime}{\mathbf{R}}^{-1}\mathbf{F}-{(\mathbf{C}}^{-1}\mathbf{W}^{\prime}{\mathbf{R}}^{-1}\mathbf{F})^{\prime}\mathbf{W}^{\prime}{\mathbf{R}}^{-1}\mathbf{F})\\=&\frac{1}{2}(\mathbf{F}{^{\prime}}{\mathbf{R}}^{-1}\mathbf{F}-{\mathbf{T}}^{\mathbf{^{\prime}}}{\mathbf{W}}^{{^{\prime}}}{\mathbf{R}}^{-1}\mathbf{F})\end{aligned}$$

where the working matrices $$\mathbf{F}=\left[\begin{array}{ccc}{\mathbf{f}}_{1}& ...& {\mathbf{f}}_{v}\end{array}\right]$$ and $$\mathbf{T}=\left[\begin{array}{ccc}{\mathbf{t}}_{1}& ...& {\mathbf{t}}_{v}\end{array}\right]$$ have *v* columns but *n* and *n*_*s*_ rows, respectively. Column *i* in **F** is [18]

9 $$\begin{aligned}{\mathbf{f}}_{i}=&\frac{\partial \mathbf{V}}{\partial {{\varvec{\uptheta}}}_{i}}\mathbf{P}\mathbf{y}\\=&\mathbf{Z}\frac{\partial \mathbf{G}}{\partial {{\varvec{\uptheta}}}_{i}}{\mathbf{G}}^{-1}\widehat{\mathbf{u}}+\frac{\partial \mathbf{R}}{\partial {{\varvec{\uptheta}}}_{i}}{\mathbf{R}}^{-1}\widehat{\mathbf{e}}\end{aligned}$$

where $\widehat{\mathbf{e}}=\mathbf{y}-\mathbf{X}\widehat{\mathbf{b}}-\mathbf{Z}\widehat{\mathbf{u}}$. Column *i* in **T** is the solution to the MME (2) but using ${\mathbf{f}}_{i}$ in place of the original **y** in the RHS (see Fig. 1 for illustration) [13]:

10 $$\mathbf{T}= {\mathbf{C}}^{-1}{\mathbf{W}}^{\prime}{\mathbf{R}}^{-1}\mathbf{F}$$

The gradient vector $\mathbf{J}\left({\varvec{\uptheta}}\right)$ element *i*, i = 1,…,*v*, is

11 $$\frac{\partial \text{log}\mathcal{L}\left({\varvec{\uptheta}}|\mathbf{y}\right)}{\partial {{\varvec{\uptheta}}}_{i}}=\frac{1}{2}tr\left(\mathbf{P}\frac{\partial \mathbf{V}}{\partial {{\varvec{\uptheta}}}_{i}}\right)-\frac{1}{2}{\mathbf{y}}^{\prime}\mathbf{P}\frac{\partial \mathbf{V}}{\partial {{\varvec{\uptheta}}}_{i}}\mathbf{P}\mathbf{y}$$

Specifically, the gradient vector for the genetic VC is

12 $$\mathbf{J}\left({{\varvec{\uptheta}}}_{\text{G}}\right)=-\frac{1}{2}vech(q{\mathbf{G}}_{0}^{-1}-{\mathbf{G}}_{0}^{-1}({\mathbf{L}}_{\text{G}}+\mathbf{U}^{\prime}{\mathbf{G}}_{\text{rm}}^{-1}\mathbf{U}){\mathbf{G}}_{0}^{-1})$$

where *q* is the number of levels within trait in the random effects **u**, i.e., the genomic breeding values in this study, **U** is a reshaped matrix of *q* by *l* of the **u** vector, and **L**_G_ is an *l* by *l* matrix. The element (*i*,*j*) in **L**_G_ is $tr\left({\mathbf{K}}_{\text{G},ij}\right)$ where ${\mathbf{K}}_{\text{G},ij}$ is an *q* by *q* submatrix of $({\mathbf{I}}_{l}\otimes {\mathbf{G}}_{\text{rm}}^{-1}){\mathbf{C}}^{\mathbf{u}\mathbf{u}}$ corresponding to the random effects of trait *i* and *j*, **C**^**uu**^ is the submatrix of ${\mathbf{C}}^{-1}$ corresponding to the random effects **u** in MME (2); ${\mathbf{I}}_{l}$ is an *l* by *l* identity matrix, and ⊗ denotes the Kronecker product.

Correspondingly, the gradient vector for the residual VC is

13 $$J\left({{\varvec{\uptheta}}}_{\text{R}}\right)=-\frac{1}{2}vech(n{\mathbf{R}}_{0}^{-1}-{\mathbf{R}}_{0}^{-1}({\mathbf{L}}_{\text{R}}+\mathbf{E}^{\prime}\mathbf{E}){\mathbf{R}}_{0}^{-1})$$

where *n* is the number of records, **E** is a reshaped matrix of residuals **e** of *n* by *l*, and **L**_R_ is an *l* by *l* matrix. The element (*i*,*j*) in **L**_R_ is $tr\left({\mathbf{K}}_{\text{R},ij}\right)$, where ${\mathbf{K}}_{\text{R},ij}$ is an *n* by *n* submatrix of ${\mathbf{W}\mathbf{C}}^{-1}{\mathbf{W}}^{\prime}$ corresponding to the traits *i* and *j*.

### The augmented AI REML

The update vector ${\varvec{\Delta}}$ of VC estimates in Eq. (4) is

14 $${\varvec{\Delta}}=-{\left[{\mathbf{I}}_{\text{A}}\left({\widehat{{\varvec{\uptheta}}}}^{\left[k-1\right]}\right)\right]}^{-1}\mathbf{J}\left({\widehat{{\varvec{\uptheta}}}}^{\left[k-1\right]}\right)$$

This indicates that **Δ** can be obtained by solving the following linear equations:

15 $$\left[{\mathbf{I}}_{\text{A}}\left({\widehat{{\varvec{\uptheta}}}}^{[k-1]}\right)\right]{\varvec{\Delta}}=-\mathbf{J}\left({\widehat{{\varvec{\uptheta}}}}^{\left[k-1\right]}\right)$$

Based on Eq. (8), Eq. (15) can be written as

16 $${\mathbf{F}}^{\mathbf{^{\prime}}}\mathbf{P}\mathbf{F}{\varvec{\Delta}}=-2\mathbf{J}\left({\widehat{{\varvec{\uptheta}}}}^{\left[k-1\right]}\right)$$

In Eq. (16), the RHS for the genetic VC (*G*_*i*_), which can be derived from Eq. (12), is

17 $$-2{\mathbf{J}\left({\varvec{\uptheta}}\right)}_{{G}_{i}}=\widehat{\mathbf{u}}\mathbf{^{\prime}}{\mathbf{G}}^{-1}(\frac{\partial \mathbf{G}}{\partial {{\varvec{\uptheta}}}_{{G}_{i}}}){\mathbf{G}}^{-1}\widehat{\mathbf{u}}-{\text{t}}_{{G}_{i}}$$

where $${\text{t}}_{{\text{G}}_{i}}=tr[{\mathbf{G}}^{-1}(\frac{\partial \mathbf{G}}{\partial {{\varvec{\uptheta}}}_{{G}_{i}}}){\mathbf{G}}^{-1}(\mathbf{G}-{\mathbf{C}}^{\mathbf{u}\mathbf{u}})]$$.

Because $\mathbf{P}\mathbf{y}= {\mathbf{R}}^{-1}\widehat{\mathbf{e}}={\mathbf{R}}^{-1}\left(\mathbf{y}-\mathbf{W}\widehat{\mathbf{s}}\right)$, with $\widehat{\mathbf{e}}=\mathbf{y}-\mathbf{W}\widehat{\mathbf{s}}$, and ${\mathbf{G}}^{-1}\widehat{\mathbf{u}}=\boldsymbol{ }{\mathbf{Z}}^{\mathbf{^{\prime}}}\mathbf{P}\mathbf{y}$ [11], then

18 $${\mathbf{G}}^{-1}\widehat{\mathbf{u}}={\mathbf{Z}}^{\mathbf{^{\prime}}}{\mathbf{R}}^{-1}\left(\mathbf{y}-\mathbf{W}\widehat{\mathbf{s}}\right)$$

Hence, Eq. (17) can be written as

19 $$-2{\mathbf{J}\left({\varvec{\uptheta}}\right)}_{{G}_{i}}={{\mathbf{f}}_{i}}^{\mathbf{^{\prime}}}{\mathbf{R}}^{-1}\left(\mathbf{y}-\mathbf{W}\widehat{\mathbf{s}}\right)-{\mathbf{t}}_{{G}_{i}}$$

because of Eq. (18) and ${\mathbf{f}}_{i}=\mathbf{Z}(\frac{\partial \mathbf{G}}{\partial {{\varvec{\uptheta}}}_{{G}_{i}}}){\mathbf{G}}^{-1}\widehat{\mathbf{u}}$ according to Eq. (9). Similarly, the RHS for the residual VC (*R*_*i*_), which can be derived from Eq. (13), is

20 $$-2{\mathbf{J}\left({\varvec{\uptheta}}\right)}_{{R}_{i}}=\widehat{\mathbf{e}}\mathbf{^{\prime}}{\mathbf{R}}^{-1}(\frac{\partial \mathbf{R}}{\partial {{\varvec{\uptheta}}}_{{R}_{i}}}){\mathbf{R}}^{-1}\widehat{\mathbf{e}}-{\text{t}}_{{R}_{i}}$$

where ${\text{t}}_{{R}_{i}}=tr[(\frac{\partial \mathbf{R}}{\partial {{\varvec{\uptheta}}}_{{R}_{i}}}){\mathbf{R}}^{-1}]-\text{tr}[(\frac{\partial \mathbf{C}}{\partial {{\varvec{\uptheta}}}_{{R}_{i}}}){\mathbf{C}}^{-1}]$. Thus, Eq. (20) can be written as

21 $$-2{\mathbf{J}\left({\varvec{\uptheta}}\right)}_{{R}_{i}}={{\mathbf{f}}_{i}}^{\mathbf{^{\prime}}}{\mathbf{R}}^{-1}\left(\mathbf{y}-\mathbf{W}\widehat{\mathbf{s}}\right)-{\mathbf{t}}_{{R}_{i}}$$

Combining Eq. (19) and Eq. (21) gives the RHS in Eq. (16) as

22 $$-2\mathbf{J}\left({\widehat{{\varvec{\uptheta}}}}^{[k-1]}\right)={\mathbf{F}}^{\mathbf{^{\prime}}}{\mathbf{R}}^{-1}\left(\mathbf{y}-\mathbf{W}\widehat{\mathbf{s}}\right)-\mathbf{t}$$

where $${\mathbf{t}}^{\mathbf{^{\prime}}}=\left[\begin{array}{cc}{\mathbf{t}}_{G}^{\mathbf{^{\prime}}}& {\mathbf{t}}_{R}^{\mathbf{^{\prime}}}\end{array}\right]$$. Thus, using Eq. (8), Eq. (15) can be expressed as

23 $$\left(\mathbf{F}\mathbf{^{\prime}}{\mathbf{R}}^{-1}\mathbf{F}-\mathbf{F}\mathbf{^{\prime}}{\mathbf{R}}^{-1}\mathbf{W}{\mathbf{C}}^{-1}\mathbf{W}\mathbf{^{\prime}}{\mathbf{R}}^{-1}\mathbf{F}\right){\varvec{\Delta}}={\mathbf{F}}^{\mathbf{^{\prime}}}{\mathbf{R}}^{-1}\left(\mathbf{y}-\mathbf{W}\widehat{\mathbf{s}}\right)-\mathbf{t}$$

Because $\mathbf{C}\widehat{\mathbf{s}}=\mathbf{W}\mathbf{^{\prime}}{\mathbf{R}}^{-1}\mathbf{y}$, the part involving $\widehat{\mathbf{s}}$ in the RHS of Eq. (23) can be reformulated:

24 $${\mathbf{F}}^{\mathbf{^{\prime}}}{\mathbf{R}}^{-1}\mathbf{W}\widehat{\mathbf{s}}={\mathbf{F}}^{\mathbf{^{\prime}}}{\mathbf{R}}^{-1}\mathbf{W}{\mathbf{C}}^{-1}\mathbf{W}\mathbf{^{\prime}}{\mathbf{R}}^{-1}\mathbf{y}$$

This allows expressing Eq. (23) in an augmented form [19] as follows:

25 $$\left[\begin{array}{cc}\mathbf{C}& \mathbf{W}\mathbf{^{\prime}}{\mathbf{R}}^{-1}\mathbf{F}\\ \mathbf{F}\mathbf{^{\prime}}{\mathbf{R}}^{-1}\mathbf{W}& \mathbf{F}\mathbf{^{\prime}}{\mathbf{R}}^{-1}\mathbf{F}\end{array}\right]\left[\begin{array}{c}\widehat{{\mathbf{s}}^{*}}\\{\varvec{\Delta}}\end{array}\right]=\left[\begin{array}{c}{\mathbf{W}}^{\mathbf{^{\prime}}}{\mathbf{R}}^{-1}\mathbf{y}\\ {\mathbf{F}}^{\mathbf{^{\prime}}}{\mathbf{R}}^{-1}\mathbf{y}-\mathbf{t}\end{array}\right]$$

Thus, the updating vector **Δ** can be solved using an augmented MME, where $\widehat{{\mathbf{s}}^{*}}$ is the new solution vector of fixed and random effects. The original MME and model are augmented by “the update effect **Δ**” with the working matrix **F** as its model matrix. RHS of the update effects, i.e., ${\mathbf{F}}^{\mathbf{^{\prime}}}{\mathbf{R}}^{-1}\mathbf{y}$, is corrected by the trace terms **t** in the first derivatives of the REML log-likelihood (see Fig. 1 for illustration).

Equation (23) allows solving the updating vector **Δ** without the need to make the augmented MME (25). Alternatively, it is possible to avoid solving the entire MME (25) by absorbing the augmented part into the original MME when employing a direct solver during each REML iteration. Thus, to obtain the solutions for **Δ**, the computational cost can be kept low by solving:

26 $${\mathbf{L}\mathbf{H}\mathbf{S}}^{*}{\varvec{\Delta}}={\mathbf{R}\mathbf{H}\mathbf{S}}^{*}$$

where

27 $${\mathbf{L}\mathbf{H}\mathbf{S}}^{*}= \mathbf{F}\mathbf{^{\prime}}{\mathbf{R}}^{-1}\mathbf{F}-\mathbf{F}\mathbf{^{\prime}}{\mathbf{R}}^{-1}\mathbf{W}{\mathbf{C}}^{-1}\mathbf{W}\mathbf{^{\prime}}{\mathbf{R}}^{-1}\mathbf{F}$$

28 $${\mathbf{R}\mathbf{H}\mathbf{S}}^{*}= \mathbf{F}\mathbf{^{\prime}}{\mathbf{R}}^{-1}\mathbf{y}-\mathbf{t}-\mathbf{F}\mathbf{^{\prime}}{\mathbf{R}}^{-1}\mathbf{W}{\mathbf{C}}^{-1}\mathbf{W}\mathbf{^{\prime}}{\mathbf{R}}^{-1}\mathbf{y}$$

and in both Eq. (27) and Eq. (28), the term $\mathbf{W}{\mathbf{C}}^{-1}\mathbf{W}\mathbf{^{\prime}}$ is precomputed. Note that the dimension of LHS* (26) is *v* by *v*, corresponding to the augmented part only.

### Data simulation

Data were simulated over 10 generations after the base population using the AlphaSimR package [22]. The simulation had five traits. The cattle species history was used for generating the base population haplotypes with an effective population size of 200. The genome consisted of 30 chromosomes. The simulated traits were determined by 900 QTL, i.e., 30 QTL per chromosome. The QTL effects for all traits were simulated from the Gamma density with shape 0.4 and scale 1.0.

After the historical population simulation, a base population of 1000 males and 1000 females was generated. The base population individuals were mated randomly, each mating producing one offspring. After the base population, the breeding population was created by selecting the top 100 males and 1000 females from the base population and the newly generated offspring to form the breeding population. The selection was based on a phenotypic index of all traits weighing them equally. Each mating in the breeding population produced one offspring: either male or female at equal numbers. In every subsequent generation, the best 100 males and 1000 females were selected from the group consisting of the current breeding population and the offspring produced by the random mating of the breeding animals.

The final pedigree consisted of 6100 females and 6100 males after simulating the breeding programme for 10 generations. In the simulation, every individual was simulated to have one observation from all correlated traits. All individuals in the pedigree were genotyped with a total number of 54,000 single nucleotide polymorphisms (SNPs). The VC used to simulate these five traits were from Nordic Cattle Genetic Evaluation (NAV) used for the evaluation of metabolic body weight (metabolic body weight during the first, second, and third lactation, stature, and carcass weight) [23]. Table 1 presents the genetic (co)variances, heritability, and genetic correlations between these five traits.

**Table 1:** Genetic (co)variances (lower triangular elements), heritability (h^2^), and genetic correlations (elements above diagonal) used in the simulation for five traits

Trait	1	2	3	4	5	h^2^
1	*27.60*	0.97	0.95	0.65	0.77	0.46
2	31.00	*37.00*	0.98	0.70	0.84	0.50
3	34.64	41.37	*48.16*	0.68	0.85	0.56
4	10.30	12.80	14.16	*9.00*	0.59	0.60
5	117.90	148.76	171.80	61.60	*847.60*	0.52

### Analyses

The multi-trait GBLUP model (1) with the general means, genetic effects, and residuals was used to fit the simulated data. The genomic relationship matrix was constructed using VanRaden’s method 1 [20] with the allele frequencies computed from the genotyped data. We investigated VC estimation as a function of the number of VC in the model, including two-trait, three-trait, four-trait, and five-trait GBLUP models, representing 6, 12, 20, and 30 VC, respectively.

Both the augmented AI-REML and the standard AI-REML methods were applied to all models. Within each REML iteration, we used the direct solving method via inverting the coefficient matrix of the MME. In addition, for the two-, three-, and four-trait GBLUP models, we utilized the PCG solver as an iterative solving method to assess the augmented AI-REML; however, for simplicity, the traces were still obtained by inversion. The convergence criteria in the PCG solver was $\Vert \mathbf{C}\mathbf{s}-\mathbf{r}\Vert <{10}^{-5}$ where **C** is the coefficient matrix of the MME, **r** is the right-hand-side vector, **s** is the solution vector, and $\Vert .\Vert$ is the Euclidean norm of a vector.

For all analyses, identity matrices were used as the initial values for the VC. The convergence indicator in both AI-REML methods was based on the relative change between the current and previous iteration of VC estimates, i.e., $\frac{{\left(\widehat{{\varvec{\uptheta}}}^{[k]} - \widehat{{\varvec{\uptheta}}}^{[k-1]}\right)^{\prime}  \left(\widehat{{\varvec{\uptheta}}}^{[k]} - \widehat{{\varvec{\uptheta}}}^{[k-1]}\right)}} {{ {\widehat{{\varvec{\uptheta}}}}^{{[k]}^{\prime}} \widehat{{\varvec{\uptheta}}}^{[k]}}}$. The threshold value was set to 1.0E-12. To compare computational efficiency, we evaluated the augmented AI-REML against the standard AI-REML in terms of the elapsed computing time per iteration. All analyses were carried out using the Julia programming language [24] on a Linux server with an Intel(R) Xeon(R) Gold 6248 CPU (2.5 GHz) and 1.5 TB RAM.

## Results

Overall, both the augmented AI-REML and the standard AI-REML produced identical VC estimates and used the same number of iterations until convergence. Table 2 shows the elapsed time per REML iteration using the direct solving method for the augmented AI-REML and the standard AI-REML across two-, three-, four-, and five-trait GBLUP models. Our analyses of the simulated datasets revealed tangible improvements in computational efficiency with the augmented AI-REML, leading to reductions in computing time per REML iteration. Although the augmented AI-REML and the standard AI-REML required similar computing times for models with a small number of VC (such as the two- and three-trait GBLUP model), the augmented AI-REML demonstrated more notable reductions in computing time as the number of VC in the model increased. The largest reduction in computing time was observed in the five-trait GBLUP model with 30 VC. Peak core memory usage was comparable between the augmented and the standard AI-REML methods.

**Table 2:** Elapsed times (seconds) for (co)variance component (VC) estimations using augmented and standard average information restricted maximum likelihood (AI-REML) with direct solver for two-, three-, four-, and five-trait genomic best linear unbiased prediction (GBLUP)

Model	Two-trait	Three-trait	Four-trait	Five-trait
ν^a^	6	12	20	30
N_eq_^b^	24,402	36,603	48,804	61,005
N_it_^c^	14	15	15	17
Standard AI-REML (s)	216	677	1554	2972
Augmented AI-REML (s)	215	674	1523	2885

^a^Number of VC in the mixed model, ^b^Number of equations in the mixed model, ^c^Number of REML iterations to achieve convergence

Table 3 presents the elapsed time per REML iteration using the iterative solving method for both the augmented AI-REML and the standard AI-REML across two-, three-, and four-trait GBLUP models. In contrast to the direct solving method, both the augmented AI-REML and the standard AI-REML exhibited longer computing times when using the iterative solving method, especially for models with many VC, such as the four-trait GBLUP model. However, the augmented AI-REML demonstrated substantial improvements in computational efficiency compared to the standard AI-REML by eliminating the need to solve the MME for each VC. Based on the analysis of the simulated datasets, the elapsed time of each REML iteration was reduced by 75%, 84%, and 86% for the two-, three-, and four-trait GBLUP models, respectively.

**Table 3:** Elapsed times (seconds) for (co)variance component (VC) estimations using augmented and standard average information restricted maximum likelihood (AI-REML) with iterative solver for two-, three-, and four-trait genomic best linear unbiased prediction (GBLUP)

Model	Two-trait	Three-trait	Four-trait
ν^a^	6	12	20
N_eq_^b^	24,402	36,603	48,804
N_it_^c^	14	15	15
Standard AI-REML (s)	2091	9780	33,452
Augmented AI-REML (s)	528	1569	3266

^a^Number of VC in the mixed model, ^b^Number of equations in the mixed model, ^c^Number of REML iterations to achieve convergence

## Discussion

In this paper, we have introduced a computationally efficient AI-REML algorithm called augmented AI-REML. While the standard AI-REML is known for its rapid convergence, it has some computational challenges, particularly when utilizing the iterative solver during AI-REML iterations. Specifically, the standard AI-REML requires solving the MME for each VC, which becomes increasingly resource-intensive as the number of VC grows. In contrast, the augmented AI-REML algorithm streamlines the computational process by solving an augmented MME only once. This novel approach offers both computational simplicity and efficiency. Notably, this study represents the first implementation of the augmented AI-REML method. Our results highlight its superiority, especially when estimating a large number of VC in the model.

A typical AI-REML algorithm relies on elements from the inverse coefficient matrix of the MME to compute the trace terms. Consequently, it has become common practice for AI-REML applications to use the direct solving method to compute the inverse. However, as the use of genomic information increases, the coefficient matrix of the MME becomes denser, posing computational challenges when analyzing large genomic datasets. Therefore, enabling VC estimation by the AI-REML method for large datasets and accelerating its computational process remains a critical concern in the genomic era. Masuda et al. [15] developed a package called YAMS. YAMS enhances the MME setup, reorders sparse structures for trace computations, and enables parallel computing for large dense blocks. They reported that the performance of YAMS was on average 10 times faster than FSPAK, a sparse matrix operation package based on traditional pedigree-based models. Laporte et al. [25] introduced the Min–Max (MM) algorithm as an alternative to the classical AI-REML algorithm for VC estimation in plant breeding. Although their method requires deriving a surrogate function within each iteration, it can offer a promising computational speed-up. Meyer [16] proposed a computational strategy involving the reparameterization of the MME to principal components. Her approach takes into account differences in the sparsity of the coefficient matrix within the single-step GBLUP model. She demonstrated a substantial reduction in computing time per iteration by leveraging this transformation to principal components.

In the current study, we first applied the direct solving method to both the augmented and the standard AI-REML. Given that the inverse of the coefficient matrix was precomputed and stored in memory, solving the MME multiple times within each iteration via multiplication with the corresponding vector on the right-hand side did not result in extreme computational expense. Consequently, based on the current datasets, the reduction in computing time per iteration using augmented AI-REML was not significantly different from the standard AI-REML (Table 2). Moreover, parallelization techniques such as OpenMP can be employed to parallelize the MME solving step when dealing with a large number of VC.

Consider a genomic model such as GBLUP used in the current study, which produces a dense coefficient matrix for the MME. When using the direct solver, the computational cost of the augmented AI-REML can be further reduced by solving a small linear system (Eq. (26)) with a size equal to the number of VC to be estimated in the model (ν). From a theoretical perspective, the estimate of computational cost in terms of the floating point operations per second (FLOPS) can be reduced from $\frac{1}{3}{(n+ \nu )}^{3}+2{(n+\nu )}^{2}$ to $\frac{1}{3}{\nu }^{3}+2{\nu }^{2}$. Note that the FLOPS for the standard AI-REML in Eq. (10) is $\nu (2{n}^{2}-n)$, where n is the number of equations in the linear mixed model. This advantage of the augmented AI-REML is due to the precomputation of $\mathbf{W}{\mathbf{C}}^{-1}\mathbf{W}\mathbf{^{\prime}}$, which can be efficiently reused when absorbing the augmented portion of the MME into the original one. However, it is crucial to recognize that this feature cannot be preserved when using an iterative solver, because no inverted coefficient matrix of the MME is available. Consequently, the entire augmented linear system (Eq. (25)) must be solved. Another noteworthy aspect of the augmented AI-REML is that, during each iteration, it avoids computing quadratic terms, such as ${\mathbf{u}}^{\boldsymbol{^{\prime}}}{\mathbf{G}}_{\text{rm}}^{-1}\mathbf{u}$ and ${\mathbf{e}}^{\boldsymbol{^{\prime}}}\mathbf{e}$ in our examples, even though the computation time for these terms is negligible.

Iterative solving methods such as PCG, in combination with iteration on data techniques [26–28], are the preferred approach for solving large MME when computing or storing the Cholesky factor of the MME is infeasible. The advantage of the iteration on data approach lies in its avoidance of explicit construction of the MME. In a study by Matilainen et al. [18], they implemented an MC algorithm within the standard AI-REML framework called MC (standard) AI-REML. In their approach, the MC samples generated from the same distribution as the original model were used to approximate PEV/PEC as equivalent to the inverse of the coefficient matrix of the MME. The MC-based REML methods improve the capability to handle large-scale VC estimation. However, it is essential to note that the MC (standard) AI-REML requires solving the MME for each VC in the model during every iteration. Consequently, the MC AI-REML method imposes a significant computational burden, especially when dealing with complex models and large datasets, such as multi-trait random regression models.

The augmented AI-REML can offer a significant advantage over the standard AI-REML in terms of computational efficiency, particularly for large multi-trait genomic models. In the standard AI-REML approach, the inverse of the MME can be used to solve the update vector of AI-REML as in Eq. (10). In addition, the inverse can also provide the PEV/PEC values required in Eq. (12) and Eq. (13). However, when an MC approach is used, the update vector and the PEV/PEC values need to be computed separately. Consequently, the standard MC AI-REML method is computationally less attractive than the EM-REML method where only one MME solving for the update is needed [18]. The augmented AI-REML can give a significant reduction in computing time in MC AI-REML because the augmented MME need to be solved only once to obtain the update vector within each REML iteration. This increases the effectiveness of MC AI-REML and will make it an attractive approach because AI-REML often converges in fewer iterations than EM-REML. As shown in Table 3, there is a substantial reduction in the computing time per REML iteration with the augmented AI-REML. This indicates the benefit of combining the MC method with the augmented AI-REML for large-scale VC estimation.

In this study, we focused on demonstrating the augmented AI-REML algorithm in analyses of a simple multi-trait GBLUP model. This algorithm can offer computational feasibility and simplicity across various models and can be integrated into existing AI-REML applications. The reduction in computing time achieved with the augmented AI-REML depends on the dimension of the dataset and the chosen model. However, it is important to recognize that the augmented AI-REML algorithm does not improve the convergence rates. In other words, it provides identical estimates and converges within the same number of iterations as the standard AI-REML. Moreover, in our analyses, even when using the PCG solver in both the augmented and the standard AI-REML algorithms, the trace terms were still derived by brute force inversion of the coefficient matrix of the MME, rather than approximated by the MC method.

## Conclusions

In this study, we introduced and demonstrated the augmented AI-REML algorithm, which is designed to improve the computational efficiency of VC estimation by AI-REML. We compared the augmented AI-REML with the standard AI-REML, employing both direct and iterative solvers in the AI-REML algorithms. In particular, the direct solver resulted in worthwhile reductions in computing time, while the iterative solver achieved significant time savings. The reductions were larger when more VC were estimated in the model. However, further research is needed to study the effect of a larger number of estimated variance components on the convergence of the iterative method for solving the augmented system. Our results underscore the potential utility of augmented AI-REML as an appealing approach for large-scale VC estimation in the genomic era.

## Data Availability

R code used to generate the simulated data and Julia code for the augmented and standard AI. REML can be found at https://github.com/hongdinggao/AI-REML.

## References

[1] Hidalgo J, Tsuruta S, Lourenco D, Masuda Y, Huang Y, Gray KA, et al. Changes in genetic parameters for fitness and growth traits in pigs under genomic selection. J Anim Sci. 2020;98:1–12. 10.1093/jas/skaa032 PMC7039409 31999338

[2] Gao H, Madsen P, Aamand GP, Thomasen JR, Sorensen AC, Jensen J. Bias in estimates of variance components in populations undergoing genomic selection: a simulation study. BMC Genomics. 2019;20:956. 31818251 10.1186/s12864-019-6323-8 PMC6902321

[3] Misztal I, Bradford HL, Lourenco DAL, Tsuruta S, Masuda Y, Legarra A, et al. Studies on inflation of GEBV in single-step GBLUP for type. Interbull Bull. 2017;51:38–42.

[4] Wiggans GR, VanRaden PM, Cooper TA. Technical note: adjustment of all cow evaluations for yield traits to be comparable with bull evaluations. J Dairy Sci. 2012;95:3444–7. 22612979 10.3168/jds.2011-5000

[5] Misztal I, Lourenco D, Legarra A. Current status of genomic evaluation. J Anim Sci. 2020;98: skaa101. 32267923 10.1093/jas/skaa101 PMC7183352

[6] Jensen J. Estimation of genetic variance in the age of genomics. J Anim Breed Genet. 2016;133:333. 27616719 10.1111/jbg.12235

[7] Patterson HD, Thompson R. Recovery of inter-block information when block sizes are unequal. Biometrika. 1971;58:545–54.

[8] Meyer K. Estimating variances and covariances for multivariate animal models by restricted maximum likelihood. Genet Sel Evol. 1991;23:67–83.

[9] Smith AB, Cullis BR, Thompson R. The analysis of crop cultivar breeding and evaluation trials: an overview of current mixed model approaches. J Agric Sci. 2005;143:449–62.

[10] Hofer A. Variance component estimation in animal breeding: a review. J Anim Breed Genet. 1998;115:247–65.

[11] Johnson DL, Thompson R. Restricted maximum likelihood estimation of variance components for univariate animal models using sparse matrix techniques and average information. J Dairy Sci. 1995;78:449–56.

[12] Gilmour AR, Thompson R, Cullis BR. Average information REML: an efficient algorithm for variance parameter estimation in linear mixed models. Biometrics. 1995;51:1440–50.

[13] Jensen J, Mäntysaari EA, Madsen P, Thompson R. Residual maximum likelihood estimation of (co)variance components in multivariate mixed linear models using average information. J Indian Soc Agric Stat. 1997;49:215–36.

[14] Misztal I, Perez-Enciso M. Sparse matrix inversion for restricted maximum likelihood estimation of variance components by expectation-maximization. J Dairy Sci. 1993;76:1479–83.

[15] Masuda Y, Aguilar I, Tsuruta S, Misztal I. Technical note: acceleration of sparse operations for average-information REML analyses with supernodal methods and sparse-storage refinements. J Anim Sci. 2015;93:4670–4. 26523559 10.2527/jas.2015-9395

[16] Meyer K. Reducing computational demands of restricted maximum likelihood estimation with genomic relationship matrices. Genet Sel Evol. 2023;55:7. 36698054 10.1186/s12711-023-00781-7 PMC9875494

[17] Matilainen K, Mantysaari EA, Lidauer MH, Stranden I, Thompson R. Employing a Monte Carlo algorithm in expectation maximization restricted maximum likelihood estimation of the linear mixed model. J Anim Breed Genet. 2012;129:457–68. 23148971 10.1111/j.1439-0388.2012.01000.x

[18] Matilainen K, Mäntysaari EA, Lidauer MH, Strandén I, Thompson R. Employing a Monte Carlo algorithm in Newton-type methods for restricted maximum likelihood estimation of genetic parameters. PLoS ONE. 2013;8: e80821. 24339886 10.1371/journal.pone.0080821 PMC3858226

[19] Thompson R. Desert island papers—a life in variance parameter and quantitative genetic parameter estimation reviewed using 16 papers. J Anim Breed Genet. 2019;136:230–42. 31247681 10.1111/jbg.12400

[20] VanRaden PM. Efficient methods to compute genomic predictions. J Dairy Sci. 2008;91:4414–23. 18946147 10.3168/jds.2007-0980

[21] Harville DA. Bayesian inference for variance components using only error contrasts. Biometrika. 1974;61:383.

[22] Chris Gaynor R, Gorjanc G, Hickey JM. AlphaSimR: an R package for breeding program simulations. G Genes Genomes Genetics. 2021;11: jkaa017. 10.1093/g3journal/jkaa017 PMC8022926 33704430

[23] Mehtiö T, Pitkänen T, Leino AM, Mäntysaari EA, Kempe R, Negussie E, et al. Genetic analyses of metabolic body weight, carcass weight and body conformation traits in Nordic dairy cattle. Animal. 2021;15: 100398. 34749067 10.1016/j.animal.2021.100398

[24] Bezanson J, Edelman A, Karpinski S, Shah VB. Julia: a fresh approach to numerical computing. SIAM Rev. 2017;59:65–98.

[25] Laporte F, Charcosset A, Mary-Huard T. Efficient ReML inference in variance component mixed models using a Min-Max algorithm. PLoS Comput Biol. 2022;18: e1009659. 35073307 10.1371/journal.pcbi.1009659 PMC8824334

[26] Misztal I, Gianola D. Indirect solution of mixed model equations. J Dairy Sci. 1987;70:716–23.

[27] Strandén I, Lidauer M. Solving large mixed linear models using preconditioned conjugate gradient iteration. J Dairy Sci. 1999;82:2779–87. 10629826 10.3168/jds.S0022-0302(99)75535-9

[28] Schaeffer LR, Kennedy BW. Computing strategies for solving mixed model equations. J Dairy Sci. 1986;69:575–9.

